# *FLT3* inhibitors as MRD-guided salvage treatment for molecular failure in *FLT3* mutated AML

**DOI:** 10.1038/s41375-023-01994-x

**Published:** 2023-08-09

**Authors:** Jad Othman, Nicola Potter, Katya Mokretar, David Taussig, Anjum Khan, Pramila Krishnamurthy, Anne-Louise Latif, Paul Cahalin, James Aries, Mariam Amer, Edward Belsham, Eibhlin Conneally, Charles Craddock, Dominic Culligan, Mike Dennis, Caroline Duncan, Sylvie D. Freeman, Caroline Furness, Amanda Gilkes, Paraskevi Gkreka, Katherine Hodgson, Wendy Ingram, Manish Jain, Andrew King, Steven Knapper, Panagiotis Kottaridis, Mary Frances McMullin, Unmesh Mohite, Loretta Ngu, Jenny O’Nions, Katharine Patrick, Tom Rider, Wing Roberts, Marianne Tang Severinsen, Neill Storrar, Tom Taylor, Nigel H. Russell, Richard Dillon

**Affiliations:** 1 Department of Medical and Molecular Genetics, King’s College London, London, England UK; 2 Guy’s and St Thomas’ NHS Foundation Trust, London, England UK; 3 Faculty of Medicine and Health, University of Sydney, Sydney, NSW Australia; 4 Cancer Genetics, Synnovis, London, England UK; 5 The Royal Marsden NHS Foundation Trust, London, England UK; 6 Leeds Teaching Hospitals NHS Trust, Leeds, England UK; 7 King’s College Hospital, London, England UK; 8 Queen Elizabeth University Hospital, Glasgow, Scotland UK; 9 Blackpool Teaching Hospitals NHS Foundation Trust, Blackpool, England UK; 10 Barts Cancer Institute, Queen Mary University of London, London, England UK; 11 University Hospital Southampton, Southampton, England UK; 12 Portsmouth Hospitals NHS Trust, Portsmouth, England UK; 13 St. James’s Hospital, Dublin, Ireland; 14 University Hospital Birmingham, Birmingham, England UK; 15 Aberdeen Royal Infirmary, Aberdeen, Scotland UK; 16 The Christie NHS Foundation Trust, Manchester, England UK; 17 Raigmore Hospital, Inverness, Scotland UK; 18 Institute of Immunology and Immunotherapy, University of Birmingham, Birmingham, Scotland UK; 19 Department of Haematology, Cardiff University, Cardiff, Wales UK; 20 Princess Royal University Hospital, London, England UK; 21 University Hospital Leicester, Leicester, England UK; 22 University Hospital Wales, Cardiff, Wales UK; 23 Addenbrooke’s Hospital, Cambridge, England UK; 24 School of Medicine, Cardiff University, Cardiff, Wales UK; 25 University College London Hospital NHS Foundation Trust, London, England UK; 26 Queen’s University, Belfast, Northern Ireland UK; 27 Singleton Hospital, Sketty, Wales UK; 28 Royal Devon & Exeter NHS Foundation Trust, Exeter, England UK; 29 Sheffield Childrens NHS Foundation Trust, Sheffield, England UK; 30 The Royal Sussex County Hospital, Brighton and Hove, England UK; 31 Great North Children’s Hospital, Newcastle upon Tyne, England UK; 32 Department of Hematology, Clinical Cancer Research Center, Aalborg University Hospital, Aalborg, Denmark; 33 NHS Lothian, Edinburgh, Scotland UK; 34 Nottingham University Hospital, Nottingham, England UK

**Keywords:** Molecularly targeted therapy, Acute myeloid leukaemia

## Abstract

Patients with *FLT3*-mutated AML have a high relapse rate and suboptimal outcomes. Many have co-mutations suitable for measurable residual disease (MRD) monitoring by RT-qPCR and those destined to relapse can be identified by high or rising levels of MRD, called molecular failure.  This provides a window for pre-emptive intervention, but there is little evidence to guide treatment. The use of *FLT3* inhibitors (FLT3i) appears attractive but their use has not yet been evaluated.  We identified 56 patients treated with FLT3i at molecular failure.  The *FLT3* mutation was an ITD in 52, TKD in 7 and both in 3. Over half of patients had previously received midostaurin. Molecular failure occurred at a median 9.2 months from diagnosis and was treated with gilteritinib (*n* = 38), quizartinib (*n* = 7) or sorafenib (*n* = 11). 60% achieved a molecular response, with 45% reaching MRD negativity. Haematological toxicity was low, and 22 patients were bridged directly to allogeneic transplant with another 6 to donor lymphocyte infusion. 2-year overall survival was 80% (95%CI 69–93) and molecular event-free survival 56% (95%CI 44–72). High-sensitivity next-generation sequencing for *FLT3*-ITD at molecular failure identified patients more likely to benefit. FLT3i monotherapy for molecular failure is a promising strategy which merits evaluation in prospective studies.

## Introduction

Mutations in the gene encoding the *FLT3* receptor tyrosine kinase are common in acute myeloid leukaemia (AML) [1] and are associated with a high rate of relapse and poor overall survival [2, 3]. While the incorporation of *FLT3* inhibitors (FLT3i) into frontline treatment has been shown to improve outcomes, over 40% of patients still relapse [4]. Outcomes after relapse remain poor even with the availability of second-generation FLT3i as salvage therapy [5], for example the median overall survival (OS) after salvage therapy with gilteritinib in the ADMIRAL study was 9.3 months, with 2-year survival of 3% in those with prior exposure to a FLT3i [6]. Similarly, in the QUANTUM-R study quizartinib treated patients had median OS 6.2 months [7]. Therapy with FLT3i at relapse is also associated with high rates of haematological toxicity which frequently requires dose reduction and treatment interruption, with grade 4 thrombocytopenia and neutropenia were seen in 30% and 27% of patients in QUANTUM-R and febrile neutropenia reported in 46% of patients in ADMIRAL [7, 8].

Improved therapeutic strategies are needed to improve these poor outcomes. Regimens combining FLT3i with venetoclax have shown somewhat higher response rates, for example the combination of gilteritinib and venetoclax showed median OS of 10 months but grade 3–4 haematological toxicity occurred in 80% of patients [9]. The triplet combination of gilteritinib, venetoclax and decitabine showed a 2 year OS of 29% in the relapsed/refractory cohort and was also associated with substantial haematological toxicity which was frequently dose limiting [10]. These combinations appear to improve *FLT3* mutational clearance in moderately sensitive assays compared with FLT3i monotherapy.

Molecular monitoring of measurable residual disease (MRD) can identify patients at high risk of relapse. While *FLT3* mutations alone are not recommended as targets for sequential monitoring due to their instability [11], many patients with *FLT3* mutated AML have a co-existing stable genetic lesion suitable for molecular monitoring including *NPM1* mutation (mut) or fusion genes (FG). For example, in the UK NCRI AML19 trial (*n* = 1705), 70% of patients with *FLT3* mutated AML (*n* = 481) had a co-occurring *NPM1*^mut^ or FG (unpublished data, Supplementary Fig. 1).

Patients destined to relapse can be reliably identified by rising levels of MRD (here called molecular failure) and this provides a potential window for pre-emptive intervention [11–13]. There is a growing body of evidence that intervening prior to haematological relapse may be associated with improved outcomes [14–18], but the optimal treatment in this situation remains undefined. Proceeding directly to transplant with detectable MRD is associated with very poor outcomes in patients with *FLT3* mutated disease [19, 20]. Targeted therapy with venetoclax appears effective in *NPM1*^mut^ patients with molecular failure, although those with concurrent *FLT3* ITD may respond poorly [21]. Salvage chemotherapy is frequently used but this is highly toxic, requires prolonged hospital admission, and has been shown to be inferior to FLT3i in patients with haematological relapse [5]. Currently no published data exist to support the use of FLT3i in the setting of molecular failure, however this is an attractive strategy as it could provide a low-toxicity outpatient-based salvage strategy, and may have the potential to re-establish an MRD negative state prior to transplant or cellular therapy, thus reducing relapse risk and improving overall survival.

Importantly, *FLT3* mutations are unstable between diagnosis and relapse, in particular almost half of patients treated with midostaurin in first line therapy do not have a *FLT3* mutation at relapse [22] implying that FLT3i salvage would not be effective in these patients. For patients with molecular failure, standard diagnostics for *FLT3* mutations are insufficiently sensitive and this factor may have limited the application of FLT3i salvage in the molecular failure setting. Recent advances in sequencing and bioinformatics now allow the detection of *FLT3* ITD with high sensitivity (here called *FLT3* ITD MRD) [23]. These assays identify a subgroup of patients at extremely high risk of relapse. Prognostic information provided by this assay appears to add to that provided by established MRD markers such as *NPM1*^mut^ [19, 24–26]. Moreover, these assays may identify patients for FLT3i salvage at the time of molecular failure.

Here, we describe outcomes of patients treated with FLT3i salvage at the time of molecular failure and retrospectively correlate these with results of *FLT3* ITD MRD testing.

## Patients and methods

### Patients

Patients were included in this study if they had been treated in the UK NCRI AML17 and AML19 studies which incorporated molecular monitoring after each cycle of treatment and for two years afterwards, or if they had received off-trial intensive induction chemotherapy with or without midostaurin and had undergone sequential MRD monitoring (Supplementary Fig. 2). The inclusion criteria were 1) *FLT3* ITD or TKD mutation at first AML diagnosis (repeat documentation of *FLT3* mutation at molecular relapse not required), 2) MRD monitoring by reverse transcription quantitative polymerase chain reaction (RT-qPCR) targeting *NPM1*^*mut*^ or FG, 3) ELN-defined molecular failure, 4) in haematologic remission at time of molecular failure (<5% blasts in bone marrow and no extramedullary disease, and 5) treatment with a single agent *FLT3* inhibitor at the time of molecular failure.

Patients potentially meeting the inclusion criteria were retrospectively identified by the UK NCRI AML molecular MRD monitoring laboratory from 27 referring hospitals, and eligibility was confirmed by the treating physician who also provided treatment and outcome data. Participating sites were requested to query their departmental and/or pharmacy databases for any patient treated with a FLT3i in haematological remission, to identify all potential patients and minimise selection bias. FLT3i were used off-label, with sorafenib and quizartinib accessed through compassionate access schemes and gilteritinib via coronavirus emergency drug supply arrangements. Patients were treated between March 2015 and August 2022. The project was approved by the Central Bristol Research Ethics Committee (22/SW/0042).

### Molecular failure definitions

Molecular failure definitions proposed by the ELN were adopted and comprised MRD > 1 copy/100 copies *ABL1* after the completion of induction and consolidation chemotherapy (molecular persistence), conversion of MRD negativity to positivity (molecular relapse), or a rise of ≥1 log_10_ in transcript levels from low-level positivity (molecular progression) [11]. Molecular relapse and progression were confirmed with a second bone marrow sample which had to show rising transcript levels. Molecular failure was based on RT-qPCR MRD of *NPM1* or FG, not *FLT3* MRD.

### MRD analyses

MRD testing for *NPM1*^mut^ and FG was performed as part of routine care in a central reference laboratory, by RT-qPCR using bone marrow aspirates with *ABL1* as a control gene as previously described [20]. Samples were run in triplicate and those with inadequate input RNA (*ABL1* cycle threshold >30) were excluded. MRD positivity was recorded where disease-related transcript amplification was detected before 40 cycles in ≥2/3 replicates according to Europe Against Cancer criteria [27]. RT-qPCR results are expressed as a copy number normalised to 10^5^ copies of *ABL1*.

High sensitivity MRD assays for *FLT3*-ITD or TKD were not available at the time of treatment with FLT3i was started. Available stored samples were retrospectively analysed for *FLT3* ITD MRD with targeted deep sequencing using a modification of the getITD protocol (Supplementary methods) [23, 25].

### Endpoints and statistical analysis

The primary objectives of the study were to describe response rates and survival with the treatment strategy. Response was assessed by sequential bone marrow RT-qPCR MRD measurement (recommended to be performed every 1-2 cycles) and was censored at the time of allo-SCT, donor lymphocyte infusion (DLI) or subsequent chemotherapy. An early response assessment was strongly recommended as a means to detect progressive disease due to the loss of *FLT3* mutation, with therapy switched for these patients.

Molecular response (MR) was defined as a ≥1 log_10_ reduction in *NPM1*^mut^ or FG transcript expression compared with the pre-treatment sample. Complete molecular response (CR_MRD-_) required MRD negativity in a sample affording technically adequate sensitivity (average *ABL1* cycle threshold <26.5). *A* ≥ 1 log_10_ rise in transcript levels was defined as molecular progression, and patients not meeting any of these criteria were designated stable disease.

Continuous variables are summarised using medians and inter-quartile range (IQR) with groups compared using the Wilcoxon rank-sum test, while categorical variables are displayed as frequencies and percentages and compared using the Chi-squared or Fisher’s exact tests. The impact of pre-treatment variables on response rate was assessed with logistic regression. Overall (OS) and relapse-free survival (RFS) were measured from the day of starting therapy, with haematological relapse or death included as RFS events. Molecular event-free survival (mEFS) was defined as time to molecular progression or molecular relapse, haematological relapse or death. Time-to-event variables were estimated using the Kaplan-Meier method and groups compared using the log-rank test. The impact of pre-treatment variables on these endpoints was analysed with Cox regression, with allogeneic transplant included as a time-dependent variable. All analyses were performed with R statistical software version 4.2.2 (R Core Development Team, Vienna, Austria).

## Results

### Patients

Fifty-six patients met the inclusion criteria and received pre-emptive salvage therapy for molecular failure with a single-agent *FLT3* inhibitor (Supplementary Fig. 2). Their median age was 51 (range 5–76). At first diagnosis, 52 (93%) had *FLT3*-ITD, 7 (14%) *FLT3*-TKD and 3 had both. The MRD marker used to diagnose molecular failure was *NPM1*^mut^ in 45 (80%) and a FG in 11 (20%) of whom five had *NUP98::NSD1*, four *DEK::NUP214*, and one each *CBFB::MYH11* and *RUNX1::RUNX1T1*.

Molecular failure occurred at a median of 9.2 months (range 0.9–31) from AML diagnosis and comprised molecular persistence in 9 (16%), molecular progression in 21 (38%) and molecular relapse in 26 (46%). Patients had received a median of 1 prior line of therapy (range 1–4), which included midostaurin in 29 (52%) and allo-SCT in 17 (30%) (Table 1). The level of MRD at the time of molecular failure ranged widely, from 2.9 to 400,000 copies/10^5^ *ABL1* (median 593 copies/10^5^ *ABL1*).

**Table 1:** Baseline characteristics.

Characteristic	*N* = 56
Female	33 (59%)
Median age	50.6
MRD marker	
* NPM1*	45 (80%)
* NUP98::NSD1*	5 (8.9%)
* DEK::NUP214*	4 (7.1%)
* CBFβ::MYH11*	1 (1.8%)
* RUNX1::RUNX1T1*	1 (1.8%)
FLT3-ITD at diagnosis	52 (93%)
FLT3-TKD at diagnosis	7 (14%)
Missing	6
MRC cytogenetic risk	
Favourable	2 (3.6%)
Intermediate	49 (88%)
Cytogenetics missing or failed	5 (8.9%)
ELN 2017 risk	
Favourable	16 (29%)
Intermediate	26 (46%)
Adverse	9 (16%)
Not able to assign	5 (8.9%)
Median months from diagnosis to molecular failure (IQR)	9.2 (5.3–13.7)
Number of prior lines of therapy	
1	36 (64%)
≥2	20 (36%)
Refractory disease	5
Relapse	15
Previous midostaurin	29 (52%)
On midostaurin at time of molecular failure	17 (30%)
Previous allo-SCT	17 (30%)
In first remission	12
In CR2 or at relapse	5
Type of molecular failure	
Molecular relapse	26 (46%)
Molecular persistence	9 (16%)
Molecular progression	21 (38%)
FLT3 inhibitor used for molecular failure	
Gilteritinib	38 (68%)
Quizartinib	7 (12%)
Sorafenib	11 (20%)

### Therapy

Thirty-eight (68%) patients were treated with gilteritinib, 7 (12%) with quizartinib and 11 (20%) with sorafenib. The gilteritinib starting dose was 120 mg in all but 3 patients (one 40 mg, two at 80 mg), and 12 patients increased to 200 mg, mostly due to a lack of response. Quizartinib was initiated at 30 mg and in all but one patient increased to 60 mg. Sorafenib dosing was more variable, with half of patients starting at 200 mg bd and increasing to 400 mg bd and the remainder starting on the higher dose. Forty-one patients have completed therapy either due to progression (20), successful bridge to transplant (12) or elective cessation (9). The median number of 28-day cycles of the FLT3 inhibitor, including those still on therapy, was 6 (range 1–43).

Data on treatment toxicity during the first 4 cycles was available for 33 patients, in whom a low rate of haematological toxicity was recorded. The median number of days per cycle of grade 4 neutropenia (<0.5 × 10^9^/L) was 0 (range 0–11) and grade 4 thrombocytopenia (<25 × 10^9^/L) also 0 (range 0–8). Only one patient required transfusions of blood and platelets, which occurred in the context of non-responding disease. The majority of the therapy was delivered as an outpatient, with only 9 of the 33 patients requiring hospitalisation at any time in the first 4 cycles, for a total of 15 admission events of median duration 3 days (range 1–23 days). Twenty-two patients were bridged directly to allo-SCT after FLT3i salvage after a median of 2.5 cycles of therapy (range 1–6) and another 6 were administered pre-emptive DLI. A further seven patients who did not achieve MR were subsequently transplanted after chemotherapy salvage.

### Molecular responses

**Fig. 1:**
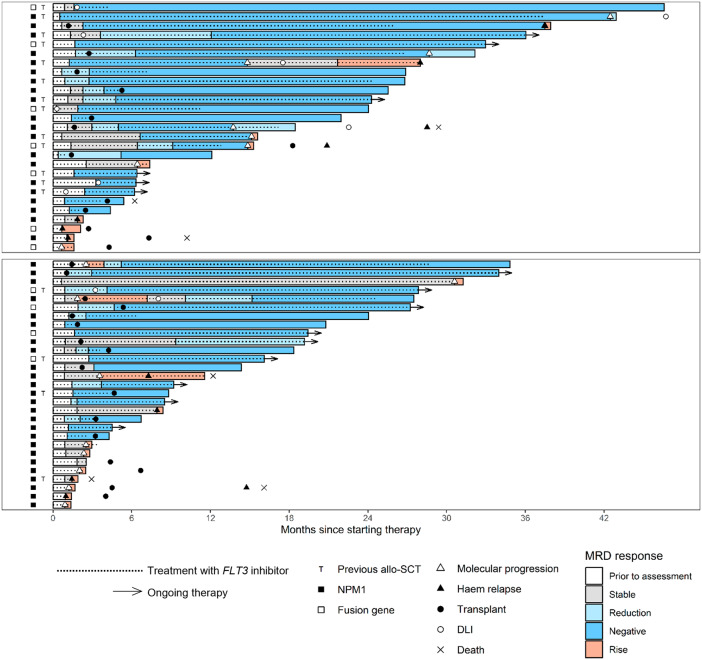
Swimmer plot of responses and events. Top panel—patients without prior FLT3 inhibitor. Bottom panel—patients with prior FLT3 inhibitor.

Response to single agent *FLT3* inhibitor salvage was assessable in 53 of 56 patients, with the first response assessment performed at a median of 44 days (range 16 - 92) after starting the FLT3i. The remaining 3 patients were administered additional therapy (DLI or allo-SCT) prior to first disease assessment. A molecular response (MR) was achieved in 32 of 53 patients (60%) and a molecular complete response (CR_MRD-_) in 24 (45%). Eight patients (17%) had stable disease and 12 (23%) progressed, including 5 with haematological relapse without preceding documented molecular progression. A swimmer plot depicting response, treatments and relapses is shown in Fig. 1.

In most responding patients there was some evidence of a response by the first assessment, 30 of 32 patients who eventually achieved at least MR showed an MRD reduction of >0.5 log_10_ on the first bone marrow assessment and 22 a >1 log_10_ decrease. The deepest MRD response achieved with *FLT3* inhibitor occurred after a median of 50 days (IQR 36 to 84). In the 28 patients who were bridged to allo-SCT or DLI, responses improved after cellular therapy, with CR_MRD-_ increasing from 48% to 96% (Supplementary Fig. 4).

**Fig. 2:**
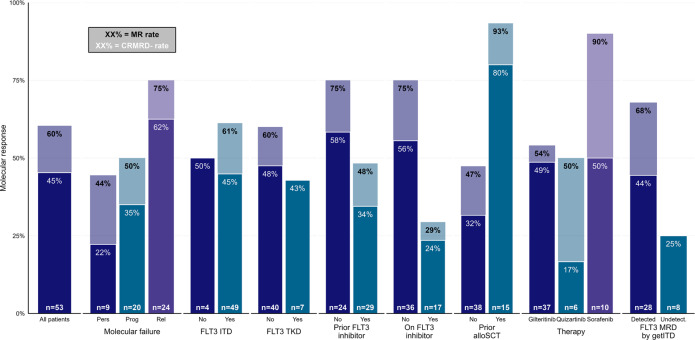
Response rates in patient subgroups. Molecular response rates by pre-treatment characteristics. Black text - overall molecular response rate. White text - CR_MRD-_ rate. Abbreviations: Pers, molecular persistence; Prog, molecular progression; Rel, molecular relapse.

Patients with *FLT3*-ITD had a response rate of 61% compared to 43% for *FLT3-*TKD (Fig. 2). In those with an *NPM1* mutation, 56% achieved MR, while eight of ten (80%) of patients with a FG had MR. Treatment at molecular relapse had a higher response rate of 75% compared to treatment at molecular progression (50%) or persistence (44%, *p* = 0.46). Patients previously exposed to midostaurin had lower response rate (48% vs 75%, *p* = 0.048), which was particularly pronounced in the subset taking midostaurin at the time of molecular failure (29% vs 75%, *p* = 0.002). There was a high response rate in patients who had received prior allo-SCT (93% vs 47% without prior allo-SCT, *p* = 0.002).

Patients treated with sorafenib had the highest rate of MR, 90% compared to 54% for gilteritinib and 50% for quizartinib. However, sorafenib was predominantly used in the post-transplant setting (7 of 11 patients) and exclusively in those without prior midostaurin exposure, limiting the utility of direct comparison between the agents.

### Outcomes

**Fig. 3:**
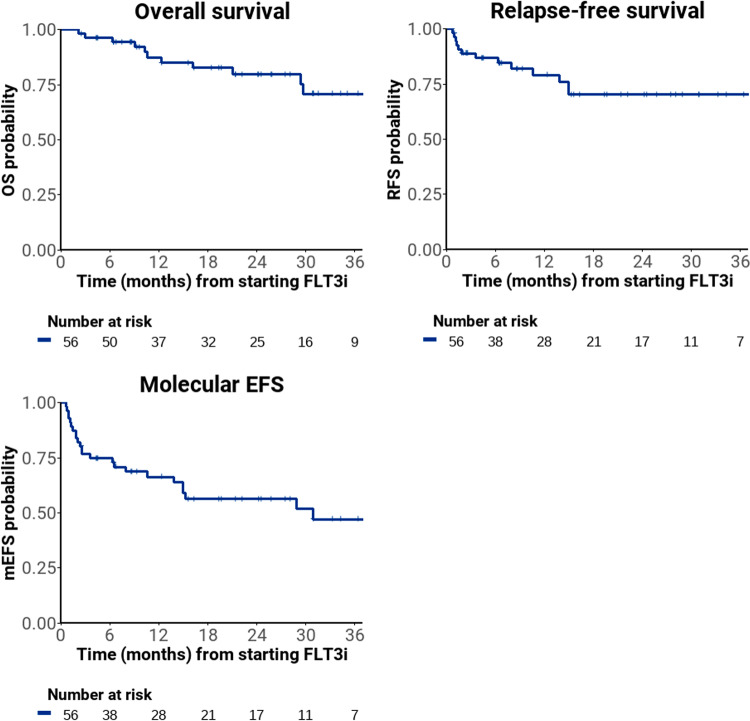
Outcomes in all patients. Overall survival, relapse-free survival and molecular event-free survival from day of starting therapy.

Median follow up by reverse Kaplan-Meier method was 25 months (95% confidence interval [CI] 21–31). At 24 months, OS was 80% (95%CI 69–93), RFS was 70% (95%CI 58–86) and mEFS was 56% (95%CI 44–72) (Fig. 3). Late relapses after stopping therapy were noted in four patients (Fig. 1). 24-month OS was 91% (95%CI 81–100) in patients who achieved MR with FLT3i monotherapy vs 60% (95%CI 41–89) in those who did not, (*p* = 0.01, Supplementary Fig. 3). Patients who were bridged to pre-emptive allo-SCT or DLI had excellent outcomes, with 2-year OS of 92% (95%CI 82–100) (Supplementary Fig. 4).

Factors associated with mEFS are shown in Table 2. On univariable analysis, older age, shorter time since diagnosis, being on midostaurin at molecular failure, no prior allo-SCT and higher MRD copy number were associated with worse mEFS. On multivariable analysis only the MRD copy number remained significant. In patients who achieved MR, allo-SCT or DLI in remission was associated with a hazard ratio for mEFS of 0.4 (95%CI 0.1–1.8, *p* = 0.2). Due to a small number of events, overall survival analyses were not possible.

**Table 2:** Univariable and multivariable Cox regression, factors impacting on molecular event-free survival.

Characteristic	Univariable			Multivariable		
	HR	95% CI	*p* value	HR	95% CI	*p* value
Age	1.03	1.00–1.06	0.02	1.03	1.00–1.06	0.06
MRD marker^a^	0.45	0.14–1.52	0.20			
FLT3-ITD at diagnosis	0.93	0.22–3.94	0.92			
Previous midostaurin	1.47	0.65–3.35	0.35			
On midostaurin at time of molecular failure	2.39	1.05–5.46	0.04	2.23	0.74–6.74	0.20
Previous allo-SCT	0.36	0.14–0.97	0.04	0.85	0.27–2.63	0.80
Molecular failure type^b^	2.15	0.93–4.94	0.07	1.28	0.51–3.22	0.60
Time between diagnosis and molecular failure	0.93	0.86–1.00	0.05	0.98	0.89–1.07	0.60
qPCR copy number at molecular failure (per 100 increase)	1.74	1.14–2.64	0.01	1.84	1.10–3.07	0.02

^a^*NPM1* as reference group.

^b^Molecular relapse as reference group, other groups combined.

### FLT3-ITD MRD analysis

Stored pre-treatment samples from 36 patients were available for *FLT3* ITD MRD. This cohort had a MR rate of 58%. *FLT3* ITD MRD was detected in the pre-treatment sample in 28/36 patients (78%), at a median VAF of 0.05%. In the 8 patients who tested *FLT3* ITD MRD negative prior to starting FLT3i, there were two molecular responses. Both responding patients had low *NPM1*^mut^ MRD copy number by qPCR (97 and 64 copies/10^5^ *ABL1*), therefore the presence of a *FLT3* ITD subclone below the lower limit of detection cannot be excluded. In the 28 patients who tested *FLT3* ITD MRD positive, MR was achieved in 18 (68%).

In samples with a low *NPM1*^mut^ /FG copy number (<200/10^5^ *ABL1*) *FLT3* ITD MRD was less likely to be detected (50% vs 88% in those with higher levels). Patients who had previously received midostaurin were more likely to test *FLT3-*ITD MRD negative at molecular failure (30% vs 12%).

*FLT3* ITD MRD testing was also performed on post-treatment samples from 39 patients, which were taken at a median 44 days (IQR 30.5–70 days) after starting therapy. The median reduction in *NPM1*^mut^/FG MRD was 1.6 log_10_ (IQR 0.2–2.4), while the median reduction in *FLT3*-ITD MRD was 1.0 log_10_ (IQR -0.2 to 1.9). All patients who had a MR by *NPM1*^mut^/FG MRD also showed a reduction in *FLT3*-ITD MRD (median reduction 1.5 log_10_, IQR 1.0–3.1). All patients not achieving a MR by *NPM1*^mut^/FG MRD, who were *FLT3* ITD MRD positive prior to therapy, demonstrated an increase in the ITD VAF (median reduction −0.7 log_10_, IQR −1.5 to −0.3, Supplementary Fig. 5). Of the non-responders who did not have a detectable ITD prior to treatment, none were found to have an emergent ITD on disease progression, suggesting that their disease was truly *FLT3-*ITD wildtype.

Samples at the time of subsequent relapse were available in six patients (five molecular relapse, one haematological relapse), of whom two relapsed while still on the *FLT3* inhibitor. Half relapsed with *FLT3* wild-type disease and the other three with the same ITD still detectable.

## Discussion

This real-world study is the first reported cohort using *FLT3* inhibition as a pre-emptive salvage strategy in patients with molecular failure but who remain in haematological remission. Response rates were high and outcomes promising, even though patients were treated prior to the availability of *FLT3* specific MRD assays. The survival outcomes of our study cannot be directly compared to those of patients treated in haematological relapse due to lead time bias and a different patient population. However the ability to bridge 50% of patients to allogeneic transplant or cellular therapy was encouraging, as this is known to be a pre-requisite for long term survival [8]. In ADMIRAL and QUANTUM-R, 26% and 32% of patients were able to be bridged to transplant [7, 8]. Haematological toxicity was notably low, possibly due to greater baseline haematological reserve when treating in haematological remission. There were few recorded episodes of grade 4 neutropenia and thrombocytopenia and infrequent requirement for hospital admission or transfusion.

A number of studies have attempted to improve outcomes for relapsed *FLT3* mutated AML by combining FLT3i with other targeted therapies [9, 10], however no comparative studies have yet been performed and results from early combination studies have shown only modest improvements in response at the cost of substantial toxicity. We suggest that intervention at the time of MRD failure may allow even better outcomes with these combination therapies by allowing treatment at higher doses with less interruption. The results of the MORPHO study (NCT02997202) assessing post-transplant gilteritinib maintenance, in particular the correlation with MRD results, may potentially provide additional support for an MRD-directed strategy.

An increasing number of publications have described outcomes of various strategies for molecular failure, although none specifically for patients with *FLT3* mutations. Intensive chemotherapy with FLAG-Ida-like regimens achieves MRD negativity in 59% [20] and 80% [16] in small cohorts of *NPM1* MRD relapse. The toxicities associated with this approach make it less appealing, especially when used in patients who remain in haematological remission. Single agent azaciditine was able to achieve molecular responses in 58% of patients in a prospective study, but only 43% in the subset with *FLT3*-ITD mutations [17]. Finally venetoclax combinations have shown promise, with molecular responses in 69% in the prospective Phase 2 VALDAC study [18] and over 80% in case series [28, 29]. However, *FLT3* mutations are a known resistance mechanism when venetoclax is used in the frontline setting [21], and indeed 43% of patients who relapsed in the VALDAC study had a detectable *FLT3*-ITD. Therefore, in patients with *FLT3* mutations who suffer molecular failure, FLT3 inhibitor-based approaches offer an attractive and highly tolerable option.

Recently three studies have demonstrated that patients with *FLT3* ITD MRD measured by NGS have extremely high rates of relapse [24–26]. Our data suggest that intervention with FLT3 inhibition with or without other targeted therapies in these patients has potential to significantly improve outcomes, and safely bridge patients to allogeneic transplant. The increasing availability of *FLT3* ITD MRD assays will allow patients to be selected for this therapy more rationally, as compared to the somewhat empiric approach used in our study, where these assays weren’t available in real time.

We recognise several limitations to our study. Patient identification and data collection were retrospective, which introduces potential selection bias, although we attempted to address this by identifying and including all eligible patients at each participating centre. Toxicity data was only available on a sub-group of patients, which introduces another potential source of reporting bias. Additionally, the choice of FLT3i was at the discretion of the treating clinician and largely dictated by drug availability at the time of molecular failure. Finally, the timing of response assessments was not standardised. Nevertheless, our data demonstrate that *FLT3* inhibitor monotherapy for molecular failure is associated with low toxicity, high rates of molecular response and encouraging overall survival. These results provide rationale for future evaluation of this strategy in prospective studies.

### Supplementary information

Supplemental appendix

## Data Availability

Access to de-identified data is available via application to the corresponding author.
