# Supplementary material for: FLT3 inhibitors as MRD-guided salvage treatment for molecular failure in FLT3 mutated AML
Source: Leukemia. 2023 Aug 9;37(10):2066–72. doi: 10.1038/s41375-023-01994-x (PMC10539160; doi:10.1038/s41375-023-01994-x)
Supplement: Supplementary file 1 — Supplemental appendix [file 41375_2023_1994_MOESM1_ESM.pdf]

FLT3 inhibitors as MRD-guided salvage treatment for molecular failure in *FLT3* mutated AML

Supplemental appendix

Contents

Supplemental methods....................2

Supplemental figure 1 – RT-qPCR MRD markers identified in patients with *FLT3* ITD or TKD in the AML19 trial (n=481) 3

Supplemental figure 2 – patient flow chart....................4

Supplemental figure 3 – OS by response to FLT3i....................5

Supplemental figure 4 – outcomes in patients bridged to alloSCT or pre-emptive DLI. 6

Supplemental figure 5 – pre and post treatment FLT3 ITD VAF in qPCR responders and non-responder....................7

References....................8

Supplemental methods

FLT3 ITD MRD assay

FLT3 ITD MRD detection was adapted from the getITD protocol(1). Genomic DNA was extracted from stored bone marrow or whole blood using the QIAamp DNA Blood Mini Kit (Qiagen) according to manufacturer's instructions. DNA was quantitated using Qubit dsDNA BR Assay kit on a Qubit 4 Fluorometer (ThermoFisher Scientific). Exons 14 and 15 of *FTL3* were amplified by PC using 500ng of input DNA, 12.5 µL 2x NEBNext Ultra II Q5 Master Mix (New England BioLabs), 0.5 µL of the 5µM primer set (forward primer TCGTCGGCAGCGTCAGATGTGTATAAGAGACAGAGCAATTTAGGTATGAAAGCCAGCTAC, reverse primer GTCTCGTGGGCTCGGAGATGTGTATAAGAGACAGACTTTCAGCATTTTGACGGCAACC) and 2 µL water. The PCR thermocycling parameters consisted of an initial denaturation step (94°C 2 min), 8 cycles of amplification (94°C 30s, 60°C 30s, 72°C 60s) and a final elongation step (72°C 10 min). PCR products were then purified using AMPure XP beads (Beckman Coulter) according to manufacturer's instructions using a bead to PCR reaction ratio of 0.9. Sample specific unique dual indexes (IDT technologies) were then incorporated using a second round of PCR amplification: 10 µL of purified first round PCR product, 12.5 µL 2x NEBNext Ultra II Q5 Master Mix (New England BioLabs), 1 µL of a unique 10 µM dual index pair and 1.5 µL water. The same thermocycling conditions described above were used for 20 cycles of PCR, followed by a second round of bead purification. The purified NGS libraries were quantified using the Qubit dsDNA BR Assay Kit. Libraries were pooled at equimolar concentration and sequenced with 20% PhiX Control (Illumina) to a high coverage (aim 1,000,000x) on an Illumina MiSeq using the Reagent Kit v2 (500-cycle). Bioinformatic detection of FLT3-ITDs was performed using the default getITD script with default settings apart from a lower minimum VAF threshold of 0.001 (-filter_ins_vaf 0.001)

No ITD was detected in any sample where the qPCR copy number was <10 / 10^5 *ABL1*, suggesting that this level of disease was below the limit of detection of the *FLT3* NGS-MRD assay. Three patients with pre-treatment samples with qPCR MRD below this level were not considered suitable for getITD analyses.

Supplemental figure 1 – RT-qPCR MRD markers identified in patients with *FLT3* ITD or TKD in the AML19 trial (n=481)

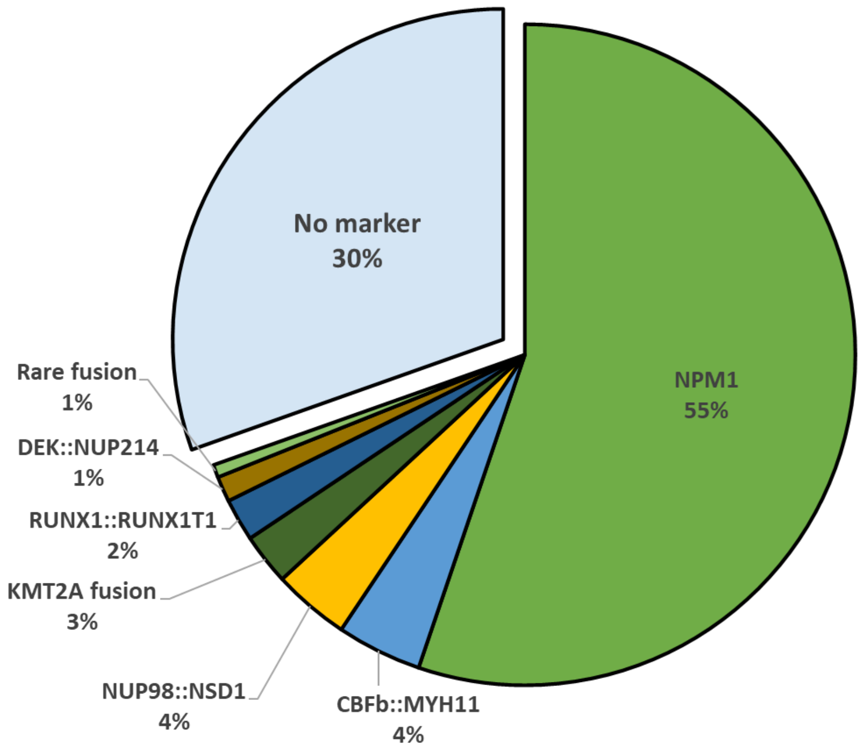

Supplemental figure 2 – patient flow chart

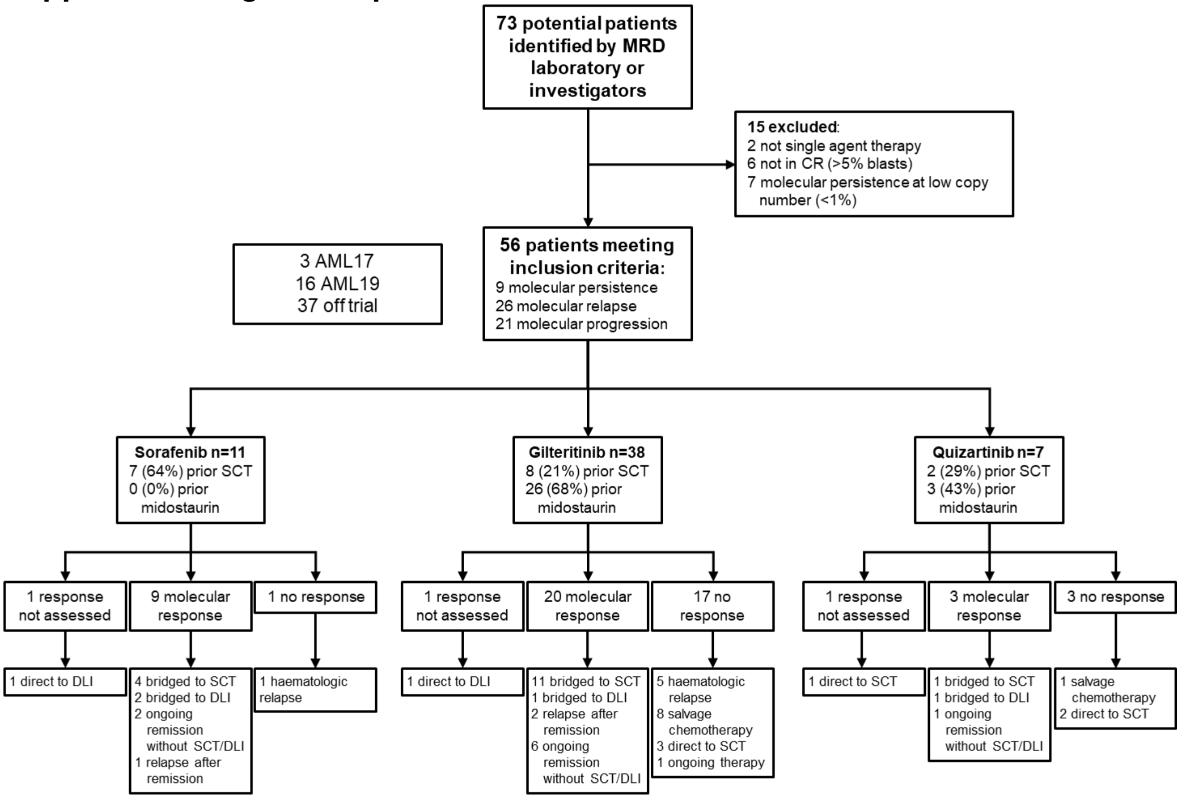

Supplemental figure 3 – OS by response to FLT3i

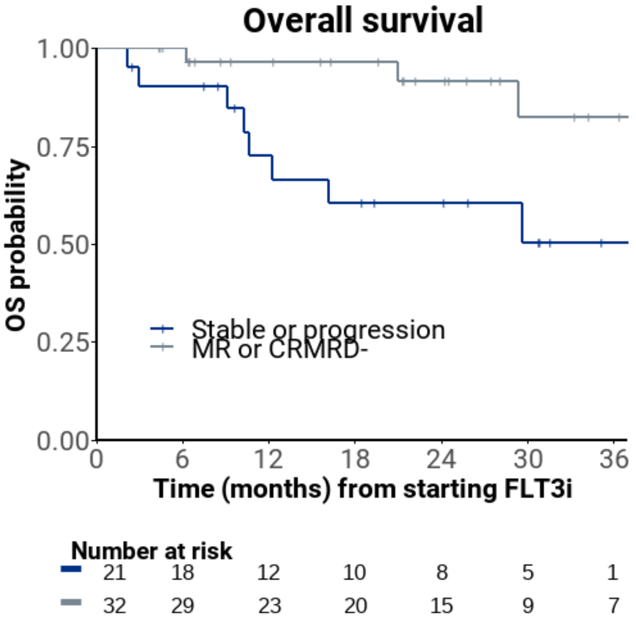

Supplemental figure 4 – outcomes in patients bridged to alloSCT or pre-emptive DLI.

A) Responses pre- and post- alloSCT/DLI

B) Overall survival from alloSCT or DLI

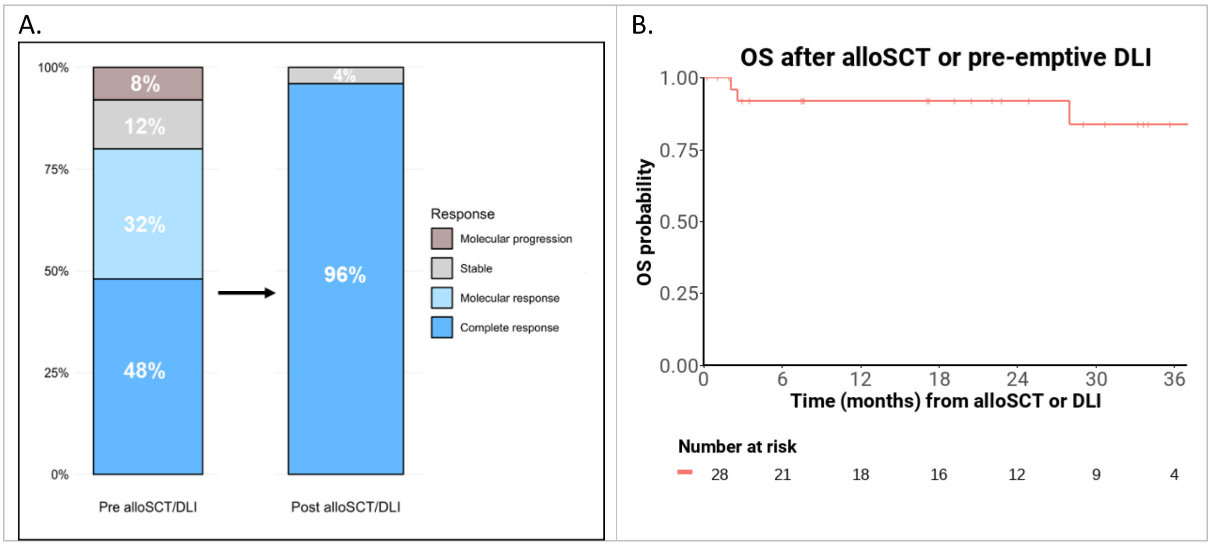

Supplemental figure 5 – pre and post treatment FLT3 ITD VAF in qPCR responders and non-responder

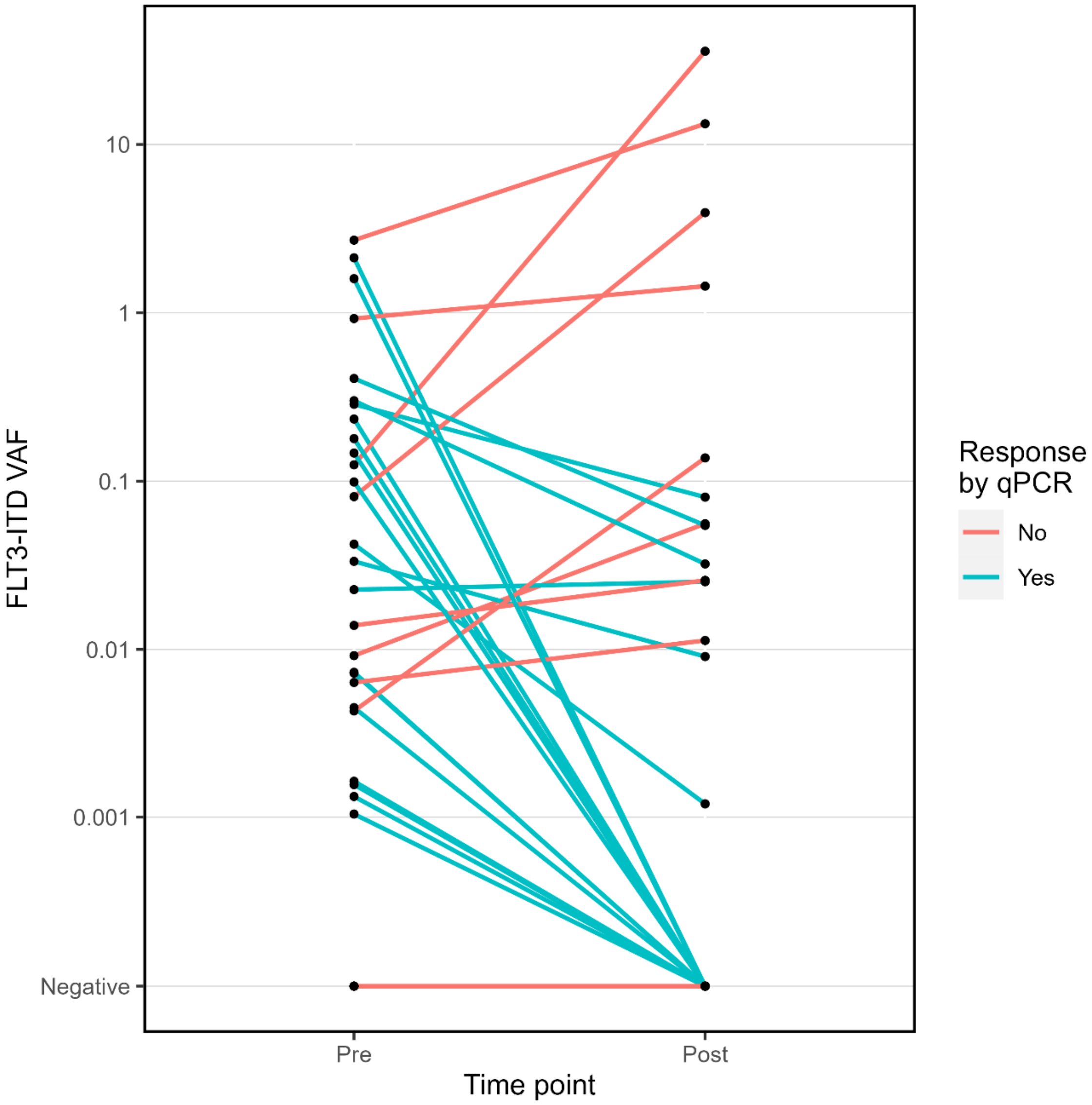

References

1. Blätte TJ, Schmalbrock LK, Skambraks S, Lux S, Cocciardi S, Dolnik A, et al. getITD for FLT3-ITD-based MRD monitoring in AML. Leukemia. 2019 Oct;33(10):2535–9.
